# The effectiveness of acupoint herbal patching for constipation after stroke: A protocol for systematic review and meta-analysis

**DOI:** 10.1097/MD.0000000000028843

**Published:** 2022-02-18

**Authors:** Yue Yuan, Ying Gao, Zihui Ding, Ying Qiao, Sanpeng Xu, Zhe Tang, Ying Liao, Ping Li

**Affiliations:** a School of Basic Medicine, Changchun University of Chinese Medicine, Changchun, China; b School of Traditional Chinese Medicine, Changchun University of Chinese Medicine, Changchun, China.

**Keywords:** acupoint herbal patching, constipation after stroke, protocol, systematic review

## Abstract

**Background:**

Constipation is one of the common problems in stroke patients, which seriously affects the life quality of patients and even leads to the recurrence of cerebrovascular disease. The clinical trials of acupoint herbal patching (AHP) in the adjuvant treatment of constipation after stroke (CAS) is currently in progress. However, there is no systematic review or meta-analysis to evaluate the effects of AHP on CAS.

**Methods:**

We will search articles in 8 electronic databases including the Cochrane Central Register of Controlled Trials, PubMed, Embase, the Web of Science, China National Knowledge Infrastructure, the Chinese Biomedical Literature Database, Wanfang Database, and the Chinese Scientific Journal Database for randomized controlled trials of CAS treated by AHP from their inception to November 1, 2021. The primary outcome measures will be clinical effective rate, defecation frequency, improvement of clinical symptoms including complete spontaneous bowel movements. The data meeting the inclusion criteria were analyzed by RevMan V.5.4 software. Two authors evaluated the study using the Cochrane collaborative risk bias tool. We will use a scoring method to assess the overall quality of evidence supporting the main results.

**Results:**

This study will analyze the clinical effective rate, defecation frequency, improvement of clinical symptoms including complete spontaneous bowel movements after stroke.

**Conclusion:**

The findings of this systematic review will provide evidence to evaluate the effectiveness and safety of AHP for CAS.

**INPLASY registration number:**

INPLASY202210065.

## 1 Introduction

Stroke is the second leading cause of death worldwide. According to the 2019 Global Burden of Disease Study, ischemic stroke accounted for 62.4%, intracerebral hemorrhage accounted for 27.9%, and subarachnoid hemorrhage accounted for 9.7%.^[1]^ Compared with the past few years, the incidence of stroke is younger, and the incidence of stroke is higher in men than in women.^[2]^ Constipation is more likely to occur when the onset site of stroke is in the basal ganglia and thalamus. Constipation after stroke (CAS) is the cause of death in 10% of stroke patients.^[3]^ The evidence from a previous systematic review shows that incidence of patients with constipation after intracerebral hemorrhage is higher than that in patients with ischemic stroke. The incidence in rehabilitation stage is higher than that in acute stage.^[4]^ The mechanism of CAS is not very clear. It is generally believed that poststroke bed rest, insufficient intake of water and cellulose, change in diet and defecation habits, and use of dehydration drugs will lead to CAS. However, in recent years, more and more evidence shows that, unlike functional constipation, poststroke constipation is more likely due to the dysfunction of brain intestinal axis after central nerve injury.^[5]^ The negative emotions caused by the need for bed rest, rehabilitation treatment and reduced self-care ability of stroke patients will enhance the rectal sensory threshold and the tension of pelvic floor muscles to a certain extent, resulting in difficult defecation.^[6]^ When patients strain to defecate, the abdominal pressure increases, causing a rapid rise in blood pressure and increasing the risk of another stroke. We should keep the stool of stroke patients unobstructed.^[7]^ Constipation is often treated with stool softeners, prokinetic agents, osmotic, and irritant laxatives, as well as lifestyle or dietary adjustment. Conventional treatment may produce adverse reactions, such as abdominal distension, dehydration, and recurrence after drug withdrawal.^[^8^,^^9^^]^ Chinese medicine method of acupoint herbal patching (AHP) is to stimulate the acupuncture points with herbs on skin directly. AHP is easy to be accepted by patients that can avoid the stimulation of the drug to the gastrointestinal tract, keep the effective components of drugs. The advantages of AHP are simplicity of operator, determined curative effect and high patient satisfaction.^[10]^ AHP treats constipation by regulating gastrointestinal motility with less adverse reactions.^[11]^ However, the clinical effective rate and safety of AHP for CAS still remain unclear, it still needs further exploration. This study aims to provide support for the clinical effective rate and safety of AHP in the treatment of CAS, which will help clinicians widely better use it in clinical treatment.

## 2 Methods and analysis

### 2.1 Design and registration information for the systematic review

This systematic review protocol has been registered with the International Platform of Registered Systematic Review and Meta-Analysis Protocols (INPLASY) and has been assigned the registration number INPLASY202210065. The study aims to evaluate the effectiveness and safety of AHP for use in the treatment CAS.

### 2.2 Types of studies

We will collect published randomized controlled trials to evaluate the clinical efficacy, functional improvement results, quality of life and adverse reactions of AHP on CAS for systematic review and meta-analysis. All eligible tests will be included regardless of language and publication type. Review articles, case series, cohort studies, retrospective studies and animal experiments that do not meet the requirements will be excluded.

### 2.3 Types of patients

Participants must have a diagnosis of stroke with symptoms of constipation without limitations related to gender, age, race, study area, and education status.

### 2.4 Types of interventions

In the intervention group patients received AHP or combined with another intervention, regardless of herbal regimen, acupoints selected, patching time.

In the control group, patients received another intervention or no treatment. Comparisons to be examined included the following.

- AHP vs no AHP.
- AHP vs another intervention.
- AHP combined with another intervention vs no AHP.

### 2.5 Types of outcome measures

#### 2.5.1 Primary outcomes

The primary outcome measures will be clinical effective rate, defecation frequency, improvement of clinical symptoms including complete spontaneous bowel movements.

#### 2.5.2 Secondary outcomes

The secondary outcomes will include adverse effects.

### 2.6 Search strategy

We will search articles in 8 electronic database including the Cochrane Central Register of Controlled Trials, PubMed, Embase, the Web of Science, China National Knowledge Infrastructure, the Chinese Biomedical Literature Database, Wanfang Database, and the Chinese Scientific Journal Database from their inception to November 1, 2021. The search string will be built as follows: (“acupoint application” or “acupoint sticker” or “herbal patch” or “herbal plaster” or “acupoint patch” or “acupoint sticking” or “point application therapy” or “drug acupoint application” or “acupuncture point application therapies” or “plaster therapy” or “external application therapy” or “acupoint herbal patching”) and (“constipation after stroke” or “difficult defecation after stroke” or “defecation disorder after stroke”) and (“randomized controlled trial” or “case-control studies” or “observational studies” or “case series” or “trial”). Reference list of all selected articles will independently screened to identify additional studies left out in the initial search. Table 1 provides details of the PubMed database search strategy. Other databases use the same search strategy. The reference list of relevant research articles was reviewed for additional article search.

**Table 1:** The search strategy for PubMed database.

Number	Search terms
#1	Acupoint application [MeSH]
#2	Acupoint sticker [MeSH]
#3	Herbal patch [MeSH]
#4	Herbal plaster [MeSH]
#5	Acupoint patch [MeSH]
#6	Acupoint sticking [MeSH]
#7	Point application therapy [MeSH]
#8	Drug acupoint application [MeSH]
#9	Acupuncture point application therapies [MeSH]
#10	Plaster therapy [MeSH]
#11	External application therapy [MeSH]
#12	Acupoint herbal patching [MeSH]
#13	OR #1-#12
#14	Constipation after stroke [MeSH]
#15	Difficult defecation after stroke [MeSH]
#16	Defecation disorder after stroke [MeSH]
#17	OR #14-#16
#18	Randomized controlled trial [MeSH]
#19	Case control studies [MeSH]
#20	Observational studies [MeSH]
#21	Case series [MeSH]
#22	Trial [MeSH]
#23	#18 or #19 or #20 or #21 or #22
#24	#13 and #17 and #23

### 2.7 Study selection and data extraction

**Figure 1:**
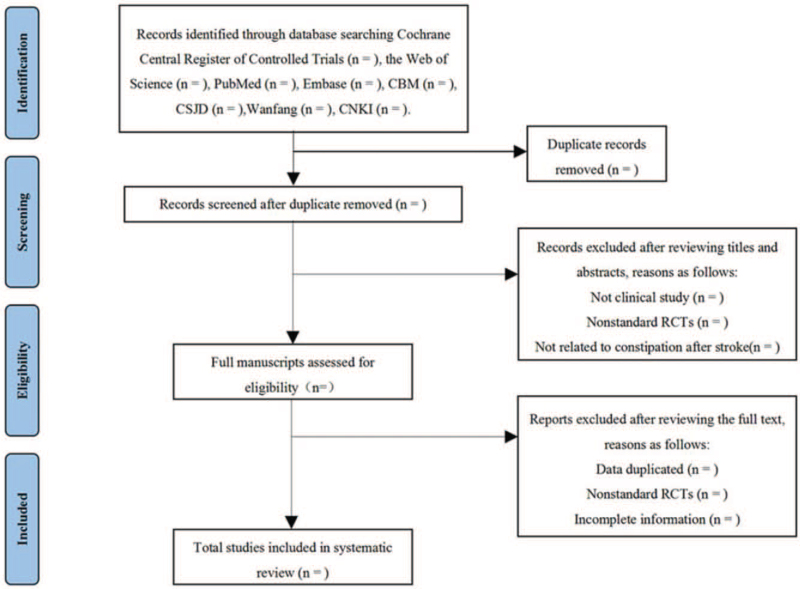
Flow chart of the search process. CBM = the Chinese Biomedical Literature Database, CNKI = the China National Knowledge Infrastructure, CSJD = the Chinese Scientific Journal Database, RCTs = randomized controlled trials.

Two review authors (YY and GY) will independently evaluate the titles and abstracts of all citations found from the above search strategy. We will obtain the full text of all potentially suitable articles to further assess eligibility based on the inclusion/exclusion criteria. Disagreements will be resolved by consensus or mediated by a third author (DZh). The final selection process will follow the PRISMA guidelines,^[12]^ as shown in Figure 1. Inclusion criteria were: participants who had CAS; AHP the intervention; randomized controlled trials; clinical effective rate, defecation frequency, functional improvement results, improvement of clinical symptoms constipation.

Exclusion criteria were: studies comparing the application, frequency or duration of different acupoints; conference abstracts without full published articles; duplicate data or data that cannot be extracted; medical history of chronic constipation or constipation caused by organic disease before stroke.

Finally, 2 authors (YY, GY) will independently extract data using data extraction tables as recommended by the Cochrane Handbook for Systematic Reviews of Interventions. The following data will be extracted: study characteristics such as author, year of publication, country in which the study was conducted, study period, original inclusion criteria, and total number of people included in the study; population characteristics such as type of CAS, mean age, and time from diagnosis; intervention characteristics such as type, duration, and frequency; outcomes such as clinical effective rate, defecation frequency, functional improvement results, improvement of clinical symptoms constipation. Any disagreements will be resolved by discussion until consensus is reached or by consulting a third researcher (DZh). We will also contact the original authors of papers via email or telephone if possible.

### 2.8 Missing data management

We will contact the study authors via email or telephone to obtain missing data and additional information if possible. Otherwise, we will analyze the available information and conduct sensitivity analysis to explore the potential impact of insufficient information on the results of meta-analysis.

### 2.9 Risk of bias assessment

Two review authors (YY and GY) will independently evaluate each included study and will follow the domain-based evaluation as developed by the Cochrane Handbook for Systematic Reviews of Interventions. They will assess the following domains: selection bias (random sequence generation and allocation concealment), performance bias (blinding of participants and personnel), detection bias (blinding of outcome assessment), attrition bias (incomplete outcome data), reporting bias (selective reporting), other bias (such as pre-sample size estimation, early stop of trial). Each domain will be divided into 3 categories: low risk of bias, unclear bias, and high risk of bias. Any discrepancies will be resolved by reviewed the original article and discussed with the third author (DZh) to reach a consensus.

### 2.10 Data synthesis and analysis

We will analyze the data with RevMan V.5.4 software (https://community.cochrane.org/help/tools-and-software/revman-5) provided by The Cochrane Collaboration. A meta-analysis using random or fixed effects models will be conducted to summarize the data. Continuous data will be pooled and presented as mean differences or standardized mean difference with their 95% CI. Dichotomous data will be pooled and expressed as risk ratio with their 95% CI. We will interpret it using the following criteria: *I* ^2^ values of 25% is considered low levels of heterogeneity, 50% indicated moderate levels, and 75% indicated high levels. Since low or moderate heterogeneity suggests little variability among these studies, the data will be analyzed in a fixed-effects model. When significant heterogeneity occurs among the studies (*P* < .05, *I* ^2^ ≥ 50%), a random effect model will be performed to analyze the data.

### 2.11 Assessment of reporting biases

Reporting bias will be assessed by Egger regression asymmetry test.^[13]^ A *P* value < .05 in Egger test is considered statistically significant. STATA V.16.0.b software (https://www.stata.com) will be used to perform the Egger test.

### 2.12 Subgroup analysis

We plan to carry out the following subgroup analyses to explore possible sources of heterogeneity: type of stroke, severity of CAS, course of disease, and treatment duration. If the data is insufficient, qualitative synthesis will be conducted instead of quantitative synthesis.

### 2.13 Sensitivity analysis

Sensitivity analysis will be performed to examine the robustness of the results by eliminating low quality trials.

### 2.14 Grading the quality of evidence

The quality of evidence regarding patient outcomes will be used assessed by the Grading of Recommendations Assessment, Development and Evaluation methodology.^[14]^ It will be used to summarize the limitations in design, consistency, directness, precision, publication bias. The quality of each evidence will be divided into 4 levels: high, medium, low, and very low. Disagreements will be resolved by consensus.

## 3 Discussion

There are dyskinesia, aphasia, dysphagia, and other dysfunction after stroke. At the same time, constipation is a common one of many complications. The clinical manifestations of constipation include persistent difficult defecation, a feeling of incomplete defecation, and a decrease in the number of defecations. Difficult defecation includes few stools, dryness, prolonged defecation process, laborious defecation, and the need to assist defecation with manual manipulation.^[15]^ Among stroke patients in rehabilitation institutions have a higher incidence of constipation, with almost all choose to use laxatives.^[16]^

At present, enema, suppositories glycerol, oral laxatives or gastrointestinal motility drugs are mainly used in clinical treatment of CAS. Enema will lead to electrolyte imbalance in patients; suppositories glycerol will increase the number of stools and weaken the normal defecation reflex, which may lead to perianal dermatitis and produce dependence; oral drugs have some problems of unstable curative effect, easy recurrence after withdrawal and poor patient compliance.^[17]^ It is urgent to find a new way of defecation that can both defecate and improve the prognosis. Nondrug treatment is the current trend.^[18]^ Traditional Chinese medicine plays an important role through its own advantages. Chinese medicinal herb, acupuncture, moxibustion, AHP, and so on are generally used to treat CAS.^[19]^ AHP is one of the characteristic therapies of traditional Chinese medicine which has the advantages of simple operation, small side effects, and low cost.

AHP is a unique external treatment of traditional Chinese medicine with the combination of acupoints and drugs based on the meridian theory, syndrome differentiation and treatment. The key targets of AHP method include the stimulation and regulation of acupoints, the pharmacodynamic effect of drugs absorbed through acupoint skin and the complementary joint action of the two.

However, there is no evidence to show the effectiveness and safety of AHP in the treatment of CAS. Therefore, this study is the first to explore the efficacy of AHP in patients with CAS through systematic review and meta-analysis. The main indicators include clinical effective rate, defecation frequency, improvement of clinical symptoms including complete spontaneous bowel movements and so on. It aims to provide clinicians with valuable references to complementary therapy and alternative therapy.

## Author contributions

Conceptualization: Yue Yuan, Ying Gao.

**Data curation:** Zihui Ding, Ying Qiao, Sanpeng Xu.

**Formal analysis:** Yue Yuan, Ying Liao, Zhe Tang.

**Funding acquisition:** Ping Li.

**Investigation:** Zhe Tang.

**Methodology:** Yue Yuan, Ying Gao.

**Project administration:** Zihui Ding, Ying Qiao.

**Resources:** Yue Yuan, Sanpeng Xu, Ying Liao, Zhe Tang.

**Software:** Yue Yuan, Ying Gao.

**Supervision:** Ping Li.

**Validation:** Ying Gao, Sanpeng Xu.

**Visualization:** Ping Li.

**Writing – original draft:** Yue Yuan.

**Writing – review & editing:** Ping Li.

## References

[1] Roth GA Mensah GA Johnson CO. Global burden of cardiovascular diseases and risk factors, 1990-2019: update from the GBD 2019 study. J Am Coll Cardiol 2020;76:2982–3021. 33309175 10.1016/j.jacc.2020.11.010 PMC7755038

[2] Wang YJ Li ZX Gu HQ. China stroke statistics 2019: a report from the national center for healthcare quality management in neurological diseases, China National Clinical Research Center for Neurological Diseases, the Chinese Stroke Association, National Center for Chronic and Non-communicable Disease Control and Prevention, Chinese Center for Disease Control and Prevention and Institute for Global Neuroscience and Stroke Collaborations. Stroke Vasc Neurol 2020;5:211–39. 32826385 10.1136/svn-2020-000457 PMC7548521

[3] Cai WZ Wang L Guo L. Analysis of related factors between constipation and brain injury after stroke. J South Med Univ 2013;33:117–20. Chinese. 23353169

[4] Li J Yuan M Liu Y Zhao Y Wang J Guo W. Incidence of constipation in stroke patients: a systematic review and meta-analysis. Medicine 2017;96:e7225. 28640117 10.1097/MD.0000000000007225 PMC5484225

[5] Camara-Lemarroy CR Ibarra-Yruegas BE Gongora-Rivera F. Gastrointestinal complications after ischemic stroke. J Neurol Sci 2014;346:20–5. 25214444 10.1016/j.jns.2014.08.027

[6] Turchina MS Bukreeva MV Korolyova LY. Correction of functional constipation in patients with limited mobility after stroke. Med Alphabet 2019;14:186–7.

[7] Higgins PD Johanson JF. Epidcmiology of constipation in North America: a systematic review. Am J Gastroenterol 2004;99:750–9. 15089911 10.1111/j.1572-0241.2004.04114.x

[8] Zhou SL Zhang XL Wang JH. Comparison of electroacupuncture and medical treatment for functional constipation: a systematic review and meta-analysis. Acupunct Med 2017;35:324–31. 28630049 10.1136/acupmed-2016-011127

[9] Zhang C Guo L Guo X. Short and long-term efficacy of combining Fuzhengliqi mixture with acupuncture in treatment of functional constipation. J Tradit Chin Med 2013;33:51–9. 23596812 10.1016/s0254-6272(13)60100-4

[10] Zhang YF. Clinical effect of acupoint application of traditional Chinese medicine on constipation after stroke of qi deficiency and blood stasis type. Clin J Tradit Chin Med 2019;31:1533–6. Chinese.

[11] Zhao H Wang SW Cao QQ. Analysis of clinical application characteristics of traditional Chinese medicine sticking method. Chin J Tradit Chin Med Pharm 2020;35:5156–9.

[12] Liberati A Altman DG Tetzlaff J. The PRISMA statement for reporting systematic reviews and meta-analyses of studies that evaluate health care interventions: explanation and elaboration. J Clin Epidemiol 2009;62:e1–34. 19631507 10.1016/j.jclinepi.2009.06.006

[13] Sterne JA Egger M Smith GD. Systematic reviews in health care: investigating and dealing with publication and other biases in meta-analysis. BMJ 2001;323:101–5. 11451790 10.1136/bmj.323.7304.101 PMC1120714

[14] Foroutan F Guyatt G Zuk V. GRADE Guidelines 28: use of GRADE for the assessment of evidence about prognostic factors: rating certainty in identification of groups of patients with different absolute risks. J Clin Epidemiol 2020;121:62–70. 31982539 10.1016/j.jclinepi.2019.12.023

[15] Sun J Min Y. Treatment progress of constipation after stroke. Chin Manipul Rehabil Med 2021;12:43–5. Chinese.

[16] Lin CJ Hung JW Cho CY. Poststroke constipation in the rehabilitation ward: incidence, clinical course and associated factors. Singapore Med J 2013;54:624–9. 24276098 10.11622/smedj.2013222

[17] Gong Zr Wang L Wang PJ. Acupoint application combined with acupuncture in the treatment of constipation after stroke. China Assoc Chin Med 2020;36:13–7. Chinese.

[18] Noergaard M Traerup AJ Jimenez-Solem E. Long term treatment with stimulant laxatives-clinical evidence for effectiveness and safety? Scand J Gastroenterol 2019;54:27–34. 30700194 10.1080/00365521.2018.1563806

[19] Yang Rd Zhou Hf. Research progress of traditional Chinese medicine in the treatment of constipation after stroke. Yunnan J Tradit Chin Med Mater Med 2019;40:88–90. Chinese.

